# Angelica Yinzi Alleviates Pruritus-Related Atopic Dermatitis through Skin Repair, Antioxidation, and Balancing Peripheral *μ*- and *κ*-opioid Receptors

**DOI:** 10.1155/2023/6058951

**Published:** 2023-09-25

**Authors:** Wei Liu, Yang Luo, Wanci Song, Hanxiong Dan, Li Li, Daonian Zhou, Pengtao You

**Affiliations:** ^1^Beijing Key Laboratory of Molecular Pharmaceutics and New Drug Delivery Systems, School of Pharmaceutical Sciences, Peking University, Beijing 100191, China; ^2^Research Center, Mayinglong Pharmaceutical Group Co. Ltd., Wuhan 430060, Hubei, China; ^3^Hubei Key Laboratory of Resources and Chemistry of Chinese Medicine, Hubei University of Chinese Medicine, Wuhan, Hubei 430065, China; ^4^Department of Pharmacy, Wuhan Hospital of Traditional Chinese Medicine, Wuhan, Hubei 430014, China

## Abstract

**Background:**

Angelica Yinzi (AYZ) is a Chinese traditional herbal formula reported to attenuate itches and inflammation caused by atopic dermatitis (AD). However, the underlying mechanism of AYZ in the attenuation of itchiness and inflammation remains unknown.

**Objective:**

This study investigated the mechanism of AYZ in reducing itchiness in mice with 1-chloro-2,4-dinitrobenzene- (DNCB-)-induced atopic dermatitis.

**Methods:**

Hematoxylin and eosin (H&E) and toluidine blue staining were used to evaluate pathological changes in skin tissue, while an enzyme‐linked immunosorbent assay (ELISA) was used to assess the cytokine levels in the skin. After that, qRT-PCR was performed to determine the mRNA levels of cytokines in the skin. Immunofluorescence and western blotting analysis were further used to assess *µ*-opioid receptor (MOR) expression and immunohistochemistry to assess the p-ERK, p-AKT, and *κ*-opioid receptor (KOR).

**Results:**

The AYZ treatment alleviated the AD clinical symptoms, including decreasing the scratching frequency, the ear thickness, and the infiltration of mast cells, lymphocytes, inflammatory cells, and mononuclear cells. In addition, AYZ inhibited the expression of interleukin (IL)-13, thymic stromal lymphopoietin (TSLP), and reduced neuraminidase (NA), corticotropin-releasing factor (CRF), and reactive oxygen species (ROS) expression. Markers involved in itches, such as p-ERK and p-AKT, were significantly downregulated following AYZ treatment. Besides, AYZ significantly increased MOR expression and downregulated KOR in the epidermis and spinal cord.

**Conclusion:**

Our findings imply that AYZ ameliorates pruritus-related AD through skin repair, antioxidation, and balancing peripheral MOR and KOR. The findings in this study lay a theoretical foundation for the control mechanism of peripheral itch.

## 1. Introduction

Atopic dermatitis (AD) is the most common chronic inflammatory skin disorder characterized by scaly red rashes, pimples, desquamation, and severe pruritus [1]. Pruritus is the most burdensome AD symptom, which, together with scratching, increases skin damage, leading to sleeplessness, fatigue, and poor life quality [2, 3]. Despite this, topical AD therapy does not control all AD symptoms, particularly pruritus, while the current systemic treatment raises a series of safety concerns.

Traditional Chinese herbal mixtures have been used to treat atopic dermatitis for many years [4–7]. Their efficacy has attracted public attention and, recently, some clinical trials have been undertaken [8–11]. Angelica Yinzi (AYZ), a traditional Chinese formula, has been used to treat skin diseases for many years, including chronic urticaria, hypersensitivity, pruritus, and AD. The AYZ formula is composed of eleven different herbs, which include Angelica Sinensis (Dang Gui), Paeoniae Radix Alba (Bai Shao), Chuanxiong Rhizoma (Chuan Xiong), Rehmannia glutinosa (Di Huang), Tribulus terrestris *L*. (Ji Li), Saposhnikoviae Radix (Fang Feng), Schizonepetae Spica (Jing Jie Sui), Polygonum multiflorum Thunb. (He Shou Wu), *Astragalus* membranaceus (Huang Qi), Glycyrrhizae Radix et Rhizoma (Gan Cao), and Ginger Root (Sheng Jiang). The AYZ formula activates several signaling pathways. For example, paeoniflorin, a major bioactive compound derived from Paeoniae Radix Alba and present in the AYZ formula, downregulates ERK1/2 and AKT phosphorylation in individuals suffering from liver disease [12]. In addition, several active ingredients in the AYZ formula effectively treat atopic dermatitis and pruritus. Moreover, the application of Angelica sinensis alleviates pruritus and skin inflammation [13, 14]. Like, Angelica gigas Nakai extract has a therapeutic effect on anti-AD [15].

Itch is considered a protective sensation and response to the environment. Scratching damages the epidermal barrier and facilitates an appropriate physiologic cascade associated with sensing the insult. This cascade includes multidirectional communication between the nervous and immune systems within the skin. Depending upon the insult, such as a contact allergen, or in association with a disease, for example, atopic dermatitis, a pathophysiologic itch may develop [15]. Pruritus is mediated in the nonmyelinated *C*-fibers whose peripheral terminals are in the skin *epidermis* and dermo-epidermal junction. Specifically, the afferent nerve fibers send signals to the second-order spinal neurons in the dorsal horn of the spinal cord, which project them to the contralateral spinothalamic tract and subsequently to the brain [16, 17]. These pruritus signals are passed on to the peripheral, spinal cord, and cortical by increasing the signals from the periphery while decreasing the neural circuits [18, 19]. Recently, the endogenous opioid system (EOS) and peripheral opioid system have been established to contribute in pruritus transmission. Besides, central nervous system neurobiological studies revealed that EOS is involved in central itch regulation [20–23]. However, it is elusive how the peripheral opioid system transmits the pruritus signals between the skin and brain.

Nevertheless, four opioid receptors have been identified, which include MOR, KOR, delta-opioid receptor (DOR), and opioid growth factor receptor-like 1 (OGFRL1) [24]. Antagonists of the *μ*-opioid receptor (MOR) and agonists of the *κ*-opioid receptor (KOR) are used to treat itch. Besides, the imbalance between MOR and KOR systems is key to pruritus development [25, 26]. Therefore, this study aimed to evaluate the therapeutic effects of AYZ against DNCB-induced AD-like symptoms in C57BL/6 mice and how the imbalance between MOR and KOR influences pruritus-related AD.

## 2. Methods

### 2.1. Chemicals and Reagents

Angelica Yinzi (AYZ) was purchased from Mayinglong Pharmaceutical Co., Ltd. (China). SDQP was purchased from Guangxi Yulin Pharmaceutical Group Co., Ltd. (China). CHT was purchased from Dong Rui Pharmaceutical Co., Ltd. (China). DNCB and olive oil were purchased from Shanghai McLean Biochemical Technology Co., Ltd. All enzyme-linked immunosorbent assay (ELISA) kits were obtained from Shanghai Fusheng Industrial Co., Ltd.

Chemical constitution analysis of AYZ using ultra-high performance liquid chromatography-quadrupole time-of-flight mass spectrometry (UHPLC/Q-TOF-MS): the UHPLC/Q-TOF-MS was used to specify the chemical compounds present in AYZ granules. Chromatographic separation was conducted on a UHPLC system (Agilent 1290 Infinity II), and the sample solution was filtered and analyzed on an Ultimate UHPLC XB-C18 column (2.1 mm × 100 mm i.d., 1.8 *μ*m). The mobile phase comprised a mixture of 0.05% formic acid and acetonitrile (*A*) and a mixture of 0.1% formic acid and pure water (*B*). The elution gradient was as follows: 0–3.5 min 5–15% *A*; 3.5–6.5 min 15–26% *A*; 6.5–7.5 min 26–27% *A*; 7.5–10 min 27–40% *A*; 10–12.5 min 40–90% *A*; 12.5–14.5 min 90% *A*; 14.5–15 min 90–5% *A*; and 15–17 min 5% *A*. The injection volume was 1 *μ*L, and the flow rate was 400 *μ*L/min.

For the qualitative analysis, the Q-TOF-MS mass spectrometer (G6530) was coupled with electrospray ionization (ESI) and a diode array detector (DAD). The samples were determined in the positive and negative modes using the following ESI parameters: dry air temperature of 350°C; dry gas flow rate of 10 L/min; atomizing gas pressure of 35 psi; sheath temperature of 350°C; sheath gas flow rate of 12 L/min; capillary voltage of 4000 V (positive mode) and 3500 V (negative mode). The Agilent MassHunter (B.08.00) was used to collect the data. Subsequently, Qualitative Navigator (B.08.00) and Qualitative Workflows (B.08.00) were used for data analysis.

### 2.2. Animals and Treatments

All animal procedures were reviewed and approved by the Animal Care and Use Committee of the Materia Medica Institute, China. Seventy-five adult male C57BL/6 mice (6 weeks old) were provided by the Hubei Provincial Center for Disease Control and Prevention (China). They were randomly assigned into five groups (*n* = 15), which were the control, model, AYZ (6.24 g/Kg), SDQP (0.96 g/Kg), and CHT groups (1.3 mg/Kg). The AD-like immunological and skin lesions were induced by treating the mice with DNCB. Briefly, 1% DNCB dissolved in acetone-olive oil (AO, 3 : 1) was applied on approximately 8 cm^2^ of dorsal skin after completely removing the hair, while 1% DNCB was applied on the face and the back of both ears after four days. All the mice except the control group were treated with 0.5% DNCB thrice weekly for three weeks (day 1–20) on the same areas on the dorsal skin. In addition, AYZ, SDQP, and CHT were dissolved in pure water and orally administered once daily for three weeks. The SDQP and CHT were used as the positive controls and are traditional Chinese medicine and western medicine, respectively, which have been accepted for the clinical treatment of AD.

On day 21, all the mice were sacrificed. The serum, lumbar (L2-L3) spinal cord contents, and lesions on the dorsal skin were collected for further analysis.

### 2.3. Evaluation of Ear Thickness and Scratching Behavior

The thicknesses of the right and left auricles were measured weekly using an electronic digital caliper during the experimental period. For elucidation, the duration of the scratching behavior was defined as the time spent rubbing the head, scratching the dorsal skin, nose, or face with the hind limbs over a 20-minute period captured using a digital camera facing the test box on the penultimate day of the experiment.

### 2.4. Cytokine Measurement

The NA, CRF, TSLP, and ROS were measured using standard ELISA kits from the serum samples according to the manufacturer's instructions. The *A* values were determined at 450 nm.

### 2.5. Histological Analysis

To investigate the effects of AYZ on DNCB-induced AD, we determined the skin thickness and mast cell infiltration. The dorsal skin samples were fixed in 4% formalin solution for 24 h, repeatedly rinsed, dehydrated, and embedded in paraffin solution. Deparaffinized skin sections were stained with H&E to determine the skin thickness and toluidine blue for mast cell infiltration.

### 2.6. Real-Time Quantitative PCR

The total RNA was extracted from the dorsal skin using the Trizol reagent (Thermo Fisher Scientific, USA) according to the manufacturer's protocol. The cDNA was then reverse-transcribed using the HiScript® III-RT SuperMix (Vazyme biotechnology, Nanjing, China). The primers were designed using the Primer Premier 5.0 design software (Premier, Canada) and synthesized by Sangon Biotech Co., Ltd. (Shanghai, China). The target gene mRNA expression levels were determined using qPCR using ChamQ Universal SYBR qPCR Master Mix (Vazyme) under the following thermocycler conditions: predegeneration at 95°C for 30 s, and 40 cycles of denaturation at 95°C for 10 s and 60°C for 30 s. The melting curve was then generated at 95°C for 15 s, 60°C for 60 s, and 95°C for 15 s. The mRNA expression was normalized using GAPDH as an internal control. The relative quantification was performed using the 2-ΔΔCT method. The primers used were:

MOR 5′-ATCCTCTCTTCTGCCATTGGT-3′, 5′-TGAAGGCGAAGATGAAGACA-3'; KOR 5′-TCCTTGGAGGCACCAAAGTCAGGG-3′, 5′-TGGTGATGCGGCGGAGATTTCG-3′; IL-13 5′-CCTGGCTCTTGCTTGCCTT-3′, 5′-GGTCTTGTGTGATGTTGCTCA-3′; Gapdh 5′-CATGGCCTTCCGTGTTCCTA-3′, 5′-CCTGCTTCACCACCTTCTTGAT-3′.

### 2.7. Immunohistochemistry

Skin, lumbar, and spinal cord samples were deparaffinized by consecutive washes with xylene and ethanol, and antigen was retrieved in citric acid buffer (pH 6.0) for 10 min. The samples were then washed in PBS for 15 min and incubated with peroxidase blocking reagent for 35 min. Next, the samples were incubated with 3% BSA at room temperature for 30 min. Subsequently, the samples were incubated with the primary antibodies, namely: p-ERK (1 : 200 dilution, Immunoway, YP1197), p-AKT (1 : 100 dilution, Abclonal, AP0140), and KOR (1 : 100 dilution, Abcam, ab183825) overnight at 4°C. Thereafter, they were incubated with the secondary horseradish peroxidase (HRP)-conjugated anti-rabbit IgG antibody (DAKO, K5007) for 50 min. The samples were then stained and visualized, and images were captured under a light microscope (Olympus, Japan).

### 2.8. Immunofluorescence

Deparaffinized skin, lumbar, and spinal cord samples were incubated with serum for 30 min, followed by overnight incubation with MOR (1 : 100 dilution, Abcam, ab135347) at 4°C. The samples were then incubated with the secondary anti-rabbit IgG antibody signed by 488 (1 : 200 dilution, Jackson 111-545-003) for 50 min. Finally, the samples were stained and visualized, and images were captured using a fluorescence microscope (Nikon, Japan).

### 2.9. Western Blotting

The skin samples were lysed in RIPA buffer and then centrifuged at 12,000 rpm for 10 min at 4°C. The proteins in the lysates were detected using a bicinchoninic acid (BCA) protein assay kit following the manufacturer's guidelines. For each sample, 100 *μ*g of protein was separated on an SDS-PAGE gel and then transferred onto nitrocellulose (NC) membranes (Millipore, Burlington, MA, USA). The membranes were blocked with 5% BSA for 2 h at room temperature, then incubated with the primary antibodies, MOR, after which they were washed in three changes of PBS. The membranes were then incubated with secondary HRP-conjugated anti-rabbit IgG antibody (1 : 200 dilution, Cell Signaling Technology, #4412) for 1 h at room temperature. Proteins were visualized by adding ECL (Thermo), then scanning and imaging the membranes using the FluorChem FC3 system (ProteinSimple, USA).

### 2.10. Statistical Analysis

All the treatments in all the assays were replicated thrice. Statistical analyses were performed using the SPSS version 22.0 statistical software. The differences among and between groups were performed by one-way analysis of variance (ANOVA) and Student's *t*-test, respectively. All data were presented as mean ± standard deviations (SD). A *p* value <0.05 was considered statistically significant.

## 3. Results

### 3.1. Quality Control Analysis of AYZ

**Figure 1:**
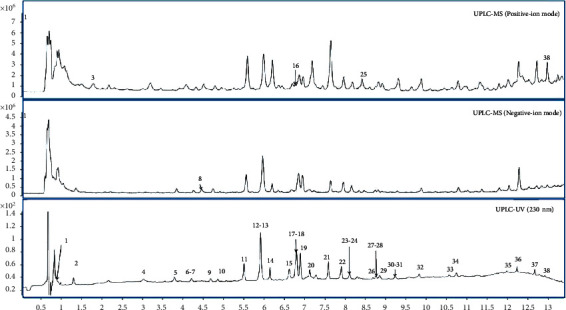
UHPLC/Q-TOF-MS base peak chromatogram of the PTQX granule extract.

The chemical ingredients of AYZ were identified by UHPLC/Q-TOF-MS in both positive and negative-ion modes. A base peak chromatogram of the PTQX granule extract was acquired for structural confirmation (Figure 1). The authentic compounds were elucidated by their MS/MS fragmentation. The quasi-molecular ions and fragment ions of compounds are listed in Table 1.

### 3.2. AYZ Attenuated DNCB-Induced AD-Like Symptoms

**Figure 2:**
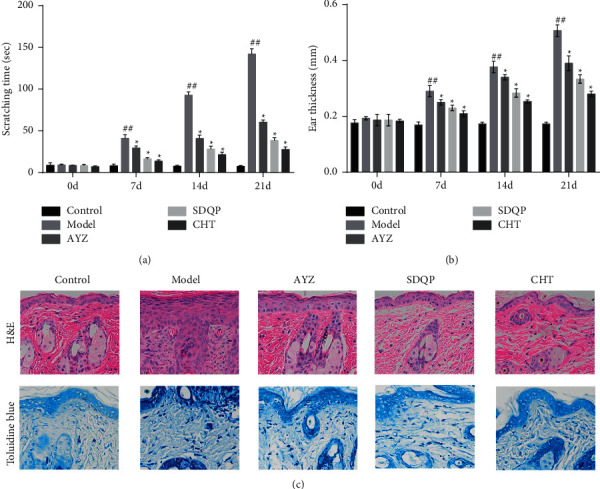
(a) Scratching frequency in the dermal lesions of C57BL/6 mice. (b) The ear thickness from day 0 to 21. (c) H&E and toluidine blue staining of mouse dorsal skin (×200). Data are expressed as the mean ± SD. ^##^*p* < 0.01, vs. the control group. ^*∗*^*p* < 0.05 vs. the model group.

Histologically, AYZ treatment significantly reduced the scratching frequency and the ear thickness (*p* < 0.05 and *p* < 0.01; shown in Figures 2(a) and 2(b)). To further examine whether AYZ inhibits DNCB-induced AD-like skin inflammation in mice, the infiltrations of inflammatory corpuscles were observed following the H&E and toluidine blue staining. There was a significant reduction in the infiltration of the mast cells, which are the major effector cells in the pathogenesis of AD, with the treatment of AYZ. Similarly, the AYZ-treated group recorded a significant reduction in lymphocytes, inflammatory cells, and mononuclear cell infiltration into dorsal skin lesions compared to the model group (shown in Figure 2(c)).

### 3.3. Effects of AYZ on DNCB-Induced Cytokine Production in Serum and ROS Level in Cutaneous Tissue

**Figure 3:**
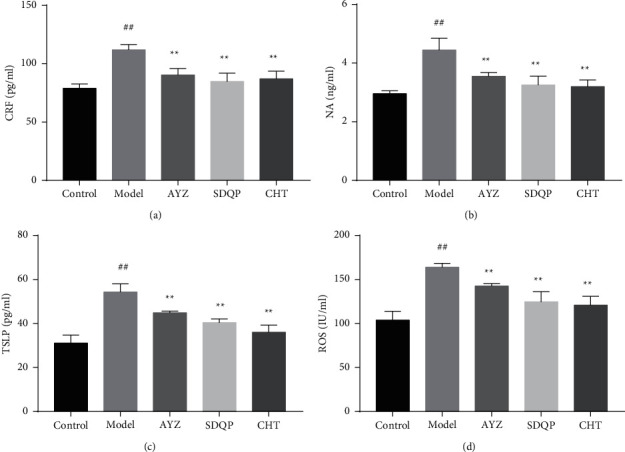
The effects of AYZ treatment on (a) CRF, (b) NA, and (c) TSLP in the serum. (d) Reactive oxygen species (ROS) in cutaneous tissue. ^##^*p* < 0.01, vs. the control group. ^*∗*^*p* < 0.05 vs. the model group.

The CRF expression levels in the model group were significantly increased compared to the control group (*p* < 0.01). In addition, the Hypothalamic-Pituitary-Adrenal (HPA) axis was activated. Moreover, cytokines were significantly downregulated following SDQP and CHT treatment. In addition, AYZ treatment significantly downregulated CRF and NA levels compared to the model group (*p* < 0.5; shown in Figure 3(a)). The AYZ treatment also decreased the expression of ceramide, a lipid degraded by NA, which maintains moisture and the skin barrier function (*p* < 0.5) (shown in Figure 3(b)). Besides, the elevated cytokine levels produced by Th2 cells in serum were also significantly decreased following AYZ treatment. However, NA was significantly increased in the DNCB-induced group compared to the control group (*p* < 0.01). Similarly, the TSLP expression levels in the DNCB group were significantly increased compared to the control group (*p* < 0.01). At the same time, the cytokines were significantly reduced following the AYZ treatment (*p* < 0.5) (shown in Figure 3(c)). The ROS quantification in cutaneous tissue revealed that the ROS levels were significantly increased in mice treated with DNCB and in the model group compared to the control group (*p* < 0.01). This implies that oxidative stress was increased in DNCB-induced AD mice. Nonetheless, AYZ treatment significantly reduced the oxidative stress levels compared to the model group (*p* < 0.5; shown in Figure 3(d)).

### 3.4. Expression of MOR and KOR Proteins in the Epidermis and Spinal Cord of Mice

**Figure 4:**
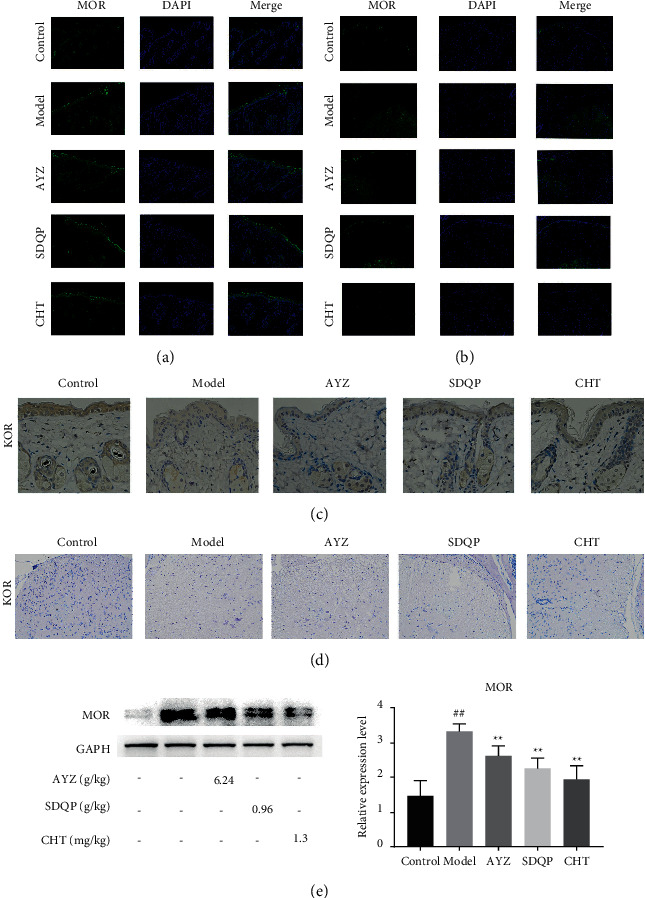
Immunofluorescence staining showing the MOR expression in the (a) *epidermis* and (b) spinal cord at ×200 magnification. Immunohistochemical staining showing the expression of KOR in the *epidermis* and spinal cord at ×200 magnification (c-d). (e) The MOR proteins expression in the *epidermis* was determined using Western blot analysis. ^##^*p* < 0.01, vs. the control group. ^*∗*^*p* < 0.05 vs. the model group.

The MOR and KOR proteins were expressed in keratinocytes present in the *epidermis*, fibroblast-like cells, and nerve fiber-like structures in the dermis. They were predominantly expressed in the upper *epidermis*. The expression of MOR was significantly enhanced (*p* < 0.01) compared to the control group, and AYZ treatment reversed the increased MOR expression in the *epidermis* and spinal cord (*p* < 0.5) (shown in Figure 4(a), 4(b)). The MOR expression in the skin was verified by western blotting (shown in Figure 4(e)). In contrast, KOR expression in the model group was significantly decreased compared to the control group (*p* < 0.01). However, the KOR expression in the *epidermis* and spinal cord was reversed following AYZ treatment compared to the model group (*p* < 0.5) (shown in Figure 4(c), 4(d)).

### 3.5. AYZ Attenuated DNCB-Induced p-ERK and p-AKT Activation in the Spinal Cord

**Figure 5:**
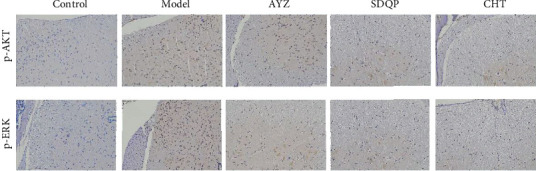
Immunohistochemical staining showing the expression of p-ERK and p-AKT in the spinal cord at ×200 magnification. ^##^*p* < 0.01, vs. the control group. ^*∗*^*p* < 0.05 vs. the model group.

To investigate the underlying mechanisms mediating the antiitch effects of AYZ, we assessed the p-ERK and p-AKT expressions in the spinal cord. The DNCB treatment significantly activated the p-ERK and p-AKT in the spinal cord, while AYZ significantly inhibited their activation compared to the control, implying that AYZ attenuated DNCB-induced p-ERK and p-AKT activation in the spinal cord (*p* < 0.5; shown in Figure 5).

### 3.6. Effects of the AYZ on IL-13, MOR, and KOR mRNA Expression in the Dorsal Skin Tissues

**Figure 6:**
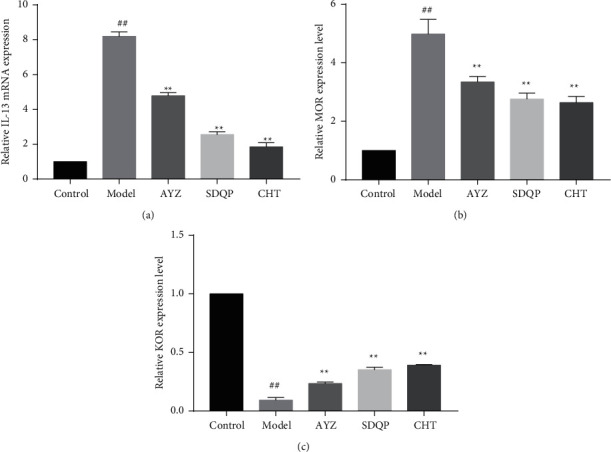
qPCR was used to evaluate IL-13, MOR, and KOR mRNA expression in DNCB-induced skin lesion tissues. ^##^*p* < 0.01, vs. the control group. ^*∗*^*p* < 0.05 vs. the model group.

Following the DNCB treatment, IL-13 and MOR mRNA levels were significantly increased while KOR mRNA expression was significantly reduced (*p* < 0.01). However, AYZ treatment successfully reversed the mRNA levels altered by DNCB treatment (*p* < 0.5) (shown in Figure 6).

## 4. Discussion

Itch is a sensation of skin discomfort that usually leads to scratching behavior in terrestrial mammals [3, 27], and this behavior can exacerbate skin damage. Itching and scratching induce sleep loss, severely affecting affected animals or individuals [26, 28]. This study revealed that AYZ alleviates pruritus-related AD by repairing the skin barrier, alleviating oxidative stress, and balancing peripheral MOR and KOR.

The TSLP and IL-13, which are key factors in AD's pathogenesis, mediate pruritus, the main AD symptom. Itch disrupts the skin barrier due to the scratching, leading to exposure to pathogens and subsequently leading to inflammatory flares [29]. However, NA plays an important role in maintaining skin permeability through its cleavage enzyme ceramide [30]. Ceramide is the main component that forms the lipid membranes between keratinocytes with water-retaining potential that prevents water loss and pathogen infection [31]. The AYZ treatment reduced the ceramidase expression, leading to skin barrier repair function.

Oxidative stress is linked to the pathogenesis of skin, systemic, and metabolic diseases [32], whose one of the symptoms is itchiness [19, 33]. However, the spinal p-ERK activation is required for acute histaminergic itch and p-AKT activation for gastrin-releasing peptide (GRP)-induced itch [34]. In the present study, AYZ reduced p-ERK and p-AKT activation in mice's spinal cord and dorsal horn, which implies that AYZ attenuated both histamine-dependent and -independent acute itch, possibly by decreasing the ROS accumulation in the skin.

Dry skin–induced pathological alterations during itchiness are processed in the peripheral nervous system and central nervous system, and subsequently manifest as exaggerated scratching behaviour, which is translated into surrogate markers of sustained stress due to the impairment of the HPA axis function [35]. Subsequently, CRF signaling leads to an opioid receptor imbalance [36, 37]. In chronic pruritic diseases, such as atopic dermatitis, lichen simplex chronicons, and prurigo simplex, the epidermal MOR is significantly unregulated, which changes the epidermal nerve fiber morphology [38]. However, the MOR and KOR knockout mice scratch less [39]. The present study established that the MOR protein levels are higher in the *epidermis* and spinal cord in AYZ-treated mice, while the KOR protein levels are lower than in control. This is consistent with a previous study where a KOR agonist, nalfurafine hydrochloride, significantly suppressed the scratching behavior [40]. Besides, Western blotting with an anti-MOR1 antibody recognizing the *N*-terminus yielded double bands, including MOR1D. Although the findings in our study did not distinguish among MOR1 isoforms, they implied that the peripheral MOR, and specifically MOR1D and KOR, were involved in itch-related behaviors in mice.

Itch is an active process initiated by MOR1D-mediated activation of GRPR [41]. GRP is an itch-specific peptide released from the primary afferents, activating the GRPR in the spinal cord in response to pruritic stimuli [42]. Spinal opiates induce itch through MOR1D and GRPR heterodimerization [41]. In addition, the effects of GRP are dependent on p-AKT activation.

The development and characterization of nalfurafine (TRK-820) for the treatment of hemodialysis-associated pruritus supports the therapeutic potential of KOR agonists to treat pruritus [43]. The KOR agonist U69,593 induces ERK1/2 and AKT phosphorylation, controlling pain perception [44].

## 5. Conclusion

In conclusion, AYZ inhibited histamine-dependent and -independent chronic itch in mice with DNCB-induced AD by skin repairs, antioxidation, and mediating the peripheral MOR and KOR balance. AYZ treatment repairs the skin barrier by reducing ceramidase expression and decreases ROS accumulation by reducing p-ERK and p-AKT activation. The activation of the HPA axis function leads to the binding of MOR1D and GRPR heterodimerization in the spinal cord. AYZ inhibits the activation of the HPA, adjusting the balance of MOR and KOR. These findings will contribute to the knowledge on effective treatment for pruritus-related AD.

## Figures and Tables

**Table 1:** Compounds detected and identified in AYZ formula.

No.	RT (min)	formula	MW (Da)	Error (ppm)	MS/MS fragments	Ion Mode	Identification	Resource
1.	0.913	C_6_H_8_O_7_	191.0197	0.14	191.0174, 111.0085, 87.0088	−	Citric acid	Pra
					59.0121			
					169.0148			
					125.0241			

2.	1.358	C_7_H_6_O_5_	169.0142	0.28	97.0298	−	Gallic acid	Pra
					79.0191			
					166.0859			
					120.0805			

3.	1.805	C_9_H_11_NO_2_	166.0862	0.33	103.0538	+	Phenylalanine	Tt, Cx, As, Rg, Ss, Gr
					205.0975			Tt, Cx, As, Ss
					188.0697			

4.	3.080	C_11_H_12_N_2_O_2_	205.0973	−0.71	170.0595	+	Tryptophan	
					146.0597			
					118.0652			Grr
					209.0465			
					165.0553			

5.	3.841	C_10_H_10_O_5_	209.0455	0.22	121.0659	−	P-hydroxybenzyl malonate	
					93.0349			
					59.0149			Pra
					495.1525			
					137.0246		Oxypaeoniflorin	Pra

6.	4.237	C_23_H_28_O_12_	495.1512	−0.81	289.0720	−		
					245.0812		Catechin	

7.	4.239	C_15_H_14_O_6_	289.0718	−0.13	125.0241	−		Ss
					159.0657			
					141.0543		2-Propylbutaned	

8.	4.465	C_7_H_12_O_4_	159.0663	−0.11	115.0763	−	Ioic acid	
					97.0661			As, Cx
					167.0348			
					123.0450		Vanillic acid	

9.	4.748	C_8_H_8_O_4_	167.0350	−0.11	79.0558	−		Grr
					417.1205			
					255.0665		Neoisoliquiritin	

10.	4.910	C_21_H_22_O_9_	417.1190	0.25	135.0086	−		Grr
					525.1620			
					479.1569		Albiflorin	

11.	5.576	C_23_H_28_O_11_	525.1614	−0.07	121.0296	−		Grr
					525.1625			
					449.1456		Paeoniflorin	

12.	5.942	C_23_H_28_O_11_	525.1614	−0.07	327.1083	−		
					121.0294			Pm
					405.1191			
					243.0659		Cis-stilbene glycoside	Sr

13.	6.017	C_20_H_22_O_9_	405.1193	−0.48	513.1622	−	Prim-O-glucosylcimifugin	
					467.1562			

14.	6.221	C_22_H_28_O_11_	513.1613	0.13	305.1022	−		
					161.0456		Ferulic acid	As, Cx
					193.0501			
					178.0269			

15.	6.670	C_10_H_10_O_4_	193.0506	0.17	160.8418	−		
					149.0605			
					134.0372		Calycosin-7-O-*β* -d-glucoside	Am
					491.1199		Liquiritin apiosid	
					283.0614			Grr

16.	6.749	C_22_H_22_O_10_	491.1196	−0.20	549.1609	−		
					417.1173			

17.	6.807	C_26_H_30_O_13_	549.1621	−1.34	255.0661	−	Liquiritin	
					135.0087			Grr
					417.1184			
					255.0659		Trans	

18.	6.861	C_21_H_22_O_9_	417.1190	0.25	135.0090	−	Stilbene glycoside	Pm
					405.1199		Cimifugin	
					243.0662			Sr

19.	6.965	C_20_H_22_O_9_	405.1190	0.26	307.1181	−		
					289.1073			

20.	7.179	C_16_H_18_O_6_	307.1176	0.05	259.0599	+	4-O-*β*-D-glucosyl-5-O-methylvisamminol	
					235.0600		Hesperidin	Sr
					497.1659			
					451.1622		Mudanpioside I	

21.	7.614	C_22_H_28_O_10_	497.1665	−0.10	271.0987	−		Ss, tt
					609.1818			
					301.0718		Rosmarinic acid	Pra

22.	7.913	C_28_H_34_O_15_	609.1829	−0.67	525.1610	−		
					479.1557			

23.	8.192	C_23_H_28_O_11_	525.1618	−0.83	121.0289	−	Senkyunolide I	Ss
					359.0775			
					197.0452		Isoliquiritin	

24.	8.194	C_18_H_16_O_8_	359.0772	0.11	161.0242	−		Cx, As
					247.0937			
					207.1010		Ononin	Grr

25.	8.402	C_12_H_16_O_4_	247.0942	−0.49	417.1179	+		
					255.0652		Rabdosiin	

26.	8.720	C_21_H_22_O_9_	417.1192	−0.23	135.0079	−		Grr, Am
					475.1236			
					267.0666		Glychionide A	Ss

27.	8.787	C_22_H_22_O_9_	475.1246	−0.03	717.1453	−		
					519.0932			

28.	8.820	C_36_H_30_O_16_	717.1462	−0.13	321.0403	−	Methylvisam minol	Grr
					445.0781			
					269.0459			

29.	8.895	C_21_H_18_O_11_	445.0774	0.53	113.0245	−	Liquiritigenin	Sr
					291.1226			
					243.0647			

30.	9.288	C_16_H_18_O_5_	291.1226	0.34	219.0648	+	sec-o-glucosylhamaudol	
					191.0696			Grr
					255.0663			
					135.0088		5R-3-Heptanone,5-hydroxy-1,7-bis	

31.	9.292	C_15_H_12_O_4_	255.0661	0.72	119.0504	−	Benzoylpaeon	Sr
					483.1517		-Iflorin	
					437.1463			

32.	9.852	C_21_H_26_O_10_	483.1509	−0.21	257.0821	−	Licorice	
					179.0554		Saponin G2	Gr
					373.1656			
					179.0716		Glycyrrhizic acid	

33.	10.634	C_21_H_26_O_6_	373.1661	−1.17		−		Ra
					629.1876			
					553.1706		6-Gingerol	

34.	10.786	C_30_H_32_O_12_	629.1876	−0.03	121.0293	−	Senkyunolide A	Grr
					837.3923			
					351.0584			

35.	12.031	C_42_H_62_O_17_	837.3914	−0.21	193.0344	−		Grr
					821.3931			
					351.0557			

36.	12.314	C_42_H_62_O_16_	821.3964	0.31	193.0348	−		Gr
								Cx
					193.1215			

37.	12.724	C_17_H_26_O_4_	294.1825	0.21	175.1110	+		

38.	12.978	C_12_H_16_O_2_	193.1221	1.07	147.1163	+		
					137.0593			

As: Angelica sinensis; Pra: Paeoniae radix alba; Cx: Chuanxiong rhizoma; Rg: Rehmannia glutinosa; Tt: Tribulus terrestris *L*; Sr: Saposhnikoviae radix; Ss: *Schizonepetae spica*; Pm: Polygonum multiflorum thunb; Am: *Astragalus* membranaceus; Grr: Glycyrrhizae radix et rhizoma; and Gr: Ginger root.

## Data Availability

The data used to support the findings of this study are available from the corresponding author upon request.
